# N^+^-Implantation on Nb Coating as Protective Layer for Metal Bipolar Plate in PEMFCs and Their Electrochemical Characteristics

**DOI:** 10.3390/ma15238612

**Published:** 2022-12-02

**Authors:** Yu-Sung Kim, Jin-Young Choi, Cheong-Ha Kim, In-Sik Lee, Shinhee Jun, Daeil Kim, Byung-Chul Cha, Dae-Wook Kim

**Affiliations:** 1 Advanced Manufacturing Process R & D Group, Ulsan Regional Division, Korea Institute of Industrial Technology (KITECH), 55, Jongga-ro, Jung-gu, Ulsan 44313, Republic of Korea; 2 DAE-IL Co., 8, Bonggyenonggong-gil, Ulju-gun, Ulsan 44914, Republic of Korea; 3 School of Materials Science & Engineering, University of Ulsan, 55-12, Techno Saneop-ro, Nam-gu, Ulsan 44776, Republic of Korea

**Keywords:** plasma immersion ion implantation, niobium, bipolar plate, corrosion resistance

## Abstract

Nitrogen ions were implanted into the coated Nb layer by plasma immersion ion implantation to improve resistance to corrosion of a metal bipolar plate. Due to nitrogen implantation, the corrosion behavior of the Nb layer was enhanced. The electron microscope observation reveals that the microstructure of the Nb layer became denser and had fewer defects with increasing implantation energy. As a result, the densified structure effectively prevented direct contact with the corrosive electrolyte. In addition, at a higher implantation rate (6.40 × 10^17^ N_2_/cm^2^), a thin amorphous layer was formed on the surface, and the implanted nitrogen ions reacted at neighboring Nb sites, resulting in the localized formation of nitrides. Such phase and structural changes contributed to further improve corrosion resistance. In particular, the implanted Nb layer at bias voltage of 10 kV exhibited a current density more than one order of magnitude smaller with a two times faster stabilization than the as-deposited Nb layer under the PEMFC operating conditions.

## 1. Introduction

To solve global warming from climate change, many countries have shown their support to the regulation of greenhouse gases from automobiles and vessels. In this regard, green vehicle technologies have been intensively researched to reduce fossil fuel consumption. Polymer electrolyte membrane fuel cells (PEMFCs) represents one of the promising candidate technologies in electric vehicles due to their higher power density, lower operating temperature, and comparatively reduced noise.

A unit cell of PEMFC stacks is composed of a membrane electrode assembly (MEA) including membrane, catalysts layers, and gas diffusion layers located between pairs of bipolar plates. Among these, the bipolar plate is one of the most important components due to its multifunctional role. The bipolar plate serves as the electrical connector between serially connected unit cells in the stack, and should be separate from fuel and oxidant.

Its other role is to provide structural supporter to the stacks, as well as water management for protecting membranes. Additionally, it should be tolerant to an aggressive acidic environment resulting from corrosive operating conditions. Therefore, the bipolar plate should provide excellent electric conductivity, gas and liquid impermeability, high mechanical properties and workability, and corrosion resistance in acidic media [1,2].

Graphite is commonly used materials in the bipolar plate because of its high electric conductivity, chemical stability, and corrosion resistance. However, graphite has a porous and brittle structure, making it difficult for machines to work on the graphite bipolar plate, which increases the production cost and time [3,4]. Thus, an alternative material with high mechanical strength and workability is needed for an effective mass production of bipolar plates to be made possible. Stainless steels have an excellent workability and mechanical properties and can be an alternative to bipolar plate. Due to higher corrosion resistance in stainless steels, 316L austenitic stainless steel (SS316L) has attracted significant attention and is widely researched [5,6,7]. However, when SS316L is exposed to highly corrosive acid environments for a long period, the metal ions from the exposed surface are dissolved, contaminating the electrolytes and catalysts and degrading performance of the PEMFC stacks.

Various surface modification techniques, including nitriding, cladding, and coating, have been applied to protect the surface of bipolar plates [8,9,10,11,12]. The nitriding process is known to be a useful method to improve the mechanical properties and corrosion resistivity of steels. However, in the case of the austenitic stainless steels, a higher treatment temperature is required to terminate the native oxide layer on the surface. This high temperature leads to the formation of CrN precipitate, which causes chromium deficiency. As a result, corrosion resistance is degraded. In another method, the cladding could be a useful modification due to this being a simple process without the need for vacuum. However, during the post annealing process, a higher temperature leads to the formation of a brittle intermetallic layer between the substrate and the cladding layer, resulting in the fracture of the intermetallic layer. Hence, a modification process that operates under low temperature is needed to prevent the above problems. The coating process, in particular physical vapor deposition, could be promising due to its ready controlling of the thickness and uniformity of the protecting layer without a high treatment temperature [11,12,13]. As a protecting material, pure niobium (Nb) possesses excellent chemical stability and corrosion resistance in an acidic environment with higher electric conductivity. Recently, we demonstrated the effect of a Nb layer on the corrosion resistance of bipolar plates through tuning microstructures. Our findings showed that the densified Nb layer with fewer defects effectively prevented direct contact between electrolyte and substrate, and exhibited good corrosion resistance in the acidic environment [14]. However, although the Nb layer was densely coated on the substrate, it could not completely eliminate inside defects including pinholes and voids, which could be a pathway for the corrosive electrolyte to penetrate. Therefore, for overcoming the above problem, additional modification of coating layer is required.

Recently, the plasma immersion ion implantation (PIII) method has beeb used as an effective way to modify the physical and chemical properties of substrate without apparent phase transformation and precipitation, i.e., improvement with maintaining its bulk properties [15,16]. A PIII process is performed at a low temperature, and physically implants ions into the substrate by adjusting the ion dose and implant ion energy. Such properties may give an advantage compared with other thermal processes. In particular, the effect of energetic ion bombardment could modify surface properties, and densify the film structure. We believe these effects could positively affect the corrosion behavior of the coated layer. Moreover, the implanted ions could contribute to further improvement in corrosion behavior. As a dopant ion, the implanted nitrogen ions (N^+^) lead to improved corrosion behavior and mechanical properties [17,18,19,20,21]. Hence, we expected that the implantation of N^+^ ions on the Nb layer would modify its structural properties and reinforce its corrosion resistance ability. In the present study, the microstructural features of the N^+^-implanted Nb layer and the interaction of implanted ions with the neighboring Nb sites were investigated. The experimental results revealed the effects of N^+^-implantation on the corrosion behavior in the simulated PEMFC environment.

## 2. Materials and Methods

A SS316L sheet with a thickness of 0.1 mm was used as the base material, and its chemical composition is shown in Table 1. In this study, the specimen was first cleaned by argon plasma etching for 30 min at 0.66 Pa. Next, a Nb layer of ca. 1.5 μm thickness was deposited on the SS316L sheet using pulsed DC magnetron sputtering in accordance with a previous study [14], as summarized in Table 2. The base pressure in the chamber was maintained at 0.001 Pa. Subsequently, N^+^ ions were implanted on the Nb-coated SS316L using PIII technique using a 60 kV class pulsed power supply, and the implantation conditions are summarized in Table 2.

In this study, both experimental and computational approaches were performed to investigate the distribution of the implanted ions. The Monte Carlo simulation program (TRIM-2013.00) was used to predict the distribution of ions. As the target material, Nb was set at 8.57 g/cm^3^ of density, and the total nitrogen ions for calculation were set at 99,999 ions. A secondary ion mass spectrometry (SIMS, IMS-6f, CAMECA, Paris, France) analysis was performed with 15 keV impact energy and 34 nA current to investigate the distribution of practically implanted ions. The phase and structure of the modified Nb layer were identified using X-ray diffraction (XRD) analysis. For this, an X-ray diffractometer (Rigaku, Tokyo, Japan, D/Max 2500-V/PC) was performed in the 2θ range of 30° to 140° with step size of 0.2°. The partial phase transformation was investigated by X-ray photoelectron spectroscopy (XPS) with a monochromic Al-Kα source (NEXSA at 15 kV, 15 mA; ThermoFisher, Waltham, MO, USA). A field emission scanning electron microscope (FE-SEM, Tokyo, Japan, JEOL JSM-5900LV) was used to examine the variation in microstructure due to the implantation of ions. The electrochemical properties were investigated using a three-electrode system by a potentiostat (Wonatech, Seoul, Republic of Korea, WPG-100). The saturated calomel electrode was used as a reference electrode, and a platinum mesh was used as a counter electrode. The PEMFC environment was simulated using 0.5 M sulfuric acid (H_2_SO_4_) aqueous solution as an electrolyte at 70 °C with saturating air as the cathode and hydrogen as the anode. The potentiodynamic curves were determined in the range of −1.5 to +1.5 V with a scan rate of 5 mV/s. In addition, a potentiostatic test was performed at the constant voltages of +0.6 V and −0.1 V with a scan rate of 1 mV/s for cathode and anode conditions, respectively, to evaluate durability in the long-term operation.

## 3. Results and Discussion

### 3.1. Range and Distribution of Implanted Ions

When ions bombard on to a target surface, since the ions randomly settle on the inside of the target, the implanted ions with the same energy or incidence angle cannot be sure to reach the same site. Thus, even though ions implant under the identical conditions, they might possibly distribute onto different site. The distributions of implanted N^+^ ions in the coated Nb layers were predicted using computational approaches, which were compared with the results from experimental approaches obtained by a secondary ion mass spectrometry (SIMS) analysis.

**Figure 1:**
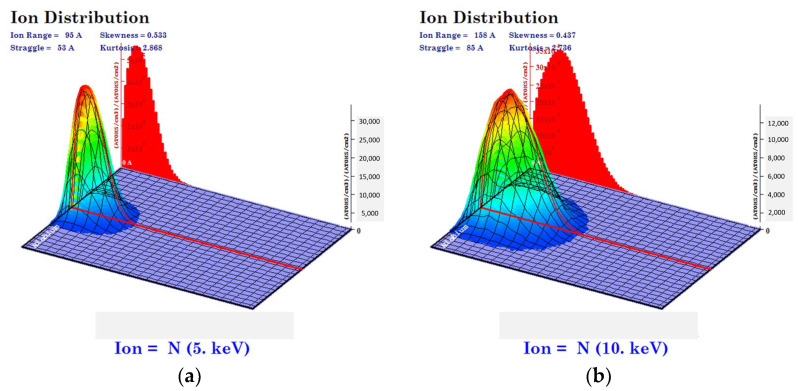
Monte Carlo simulation program (TRIM) simulations for implanted nitrogen profiles in the Nb target at the bias voltage (**a**) 5 kV and (**b**) 10 kV.

**Figure 2:**
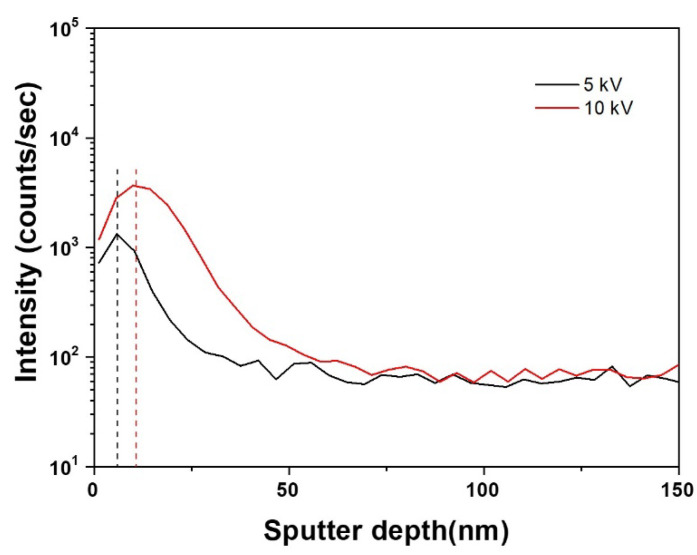
Depth profiles of nitrogen contents in the N^+^-implanted samples by secondary ion mass spectrometry (SIMS).

As shown in the simulated results (Figure 1), the depth and width of implantation tended to increase with increasing applied bias voltages. According to distribution theory for implanted ions, higher ion energy induces a wider range of ion distribution [22,23]. However, as the bias voltages increased, the peak nitrogen concentration migrated from the surface into the inside of the Nb layer with an ever increasing in implantation depth. Thus, although the total ion range and concentration increased, the nitrogen concentration at the surface decreased. The obtained results imply that a higher bias voltage induces a relatively deficient concentration of N^+^ ions at the surface, resulting in an insufficient surface modification of the Nb layer. Comparing the computational and the experimental results (Figure 2, Table 3), the implantation depth and the peak nitrogen concentration presented a similar tendency. Although 10 kV of bias voltage led to the peak nitrogen concentration migrating into the inside, the number of nitrogen ions at the surface presented a higher value than the 5 kV applied to the sample. Therefore, the obtained results suggest that if 10 kV was applied to the sample, there would be sufficiently implanted N^+^ ions at both surface and inside of the Nb layer.

### 3.2. Phase Characterization

**Figure 3:**
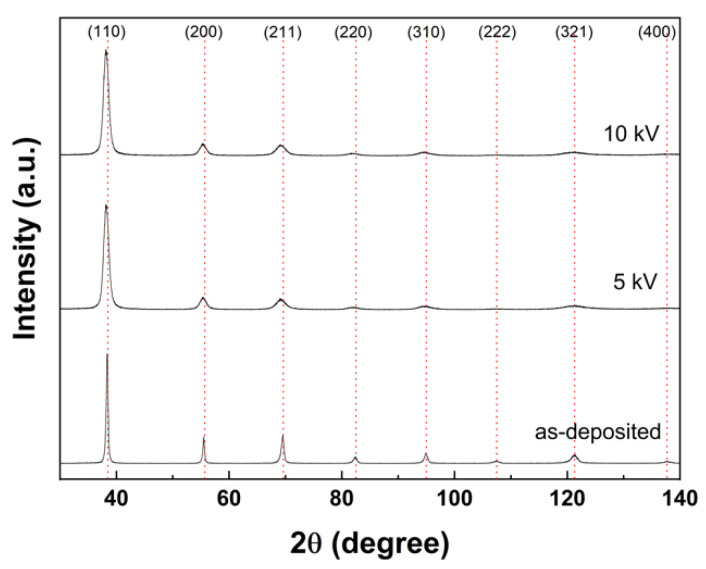
XRD patterns of the as-deposited Nb layer and N^+^-implanted samples.

**Figure 4:**
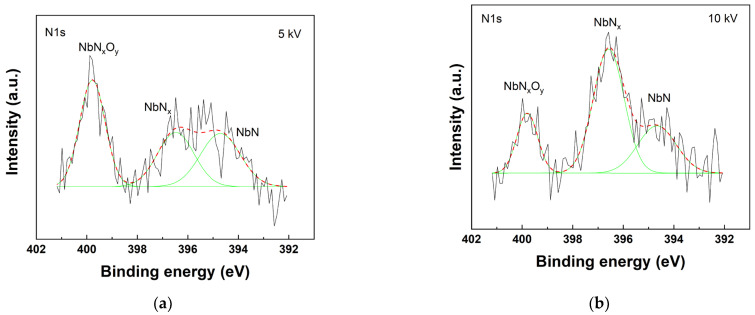
N1s XPS core level spectra of implanted N^+^ at (**a**) 5 kV and (**b**) 10 kV.

The XRD patterns were compared to investigate the effect of implantation of ions on the crystal phase, as shown in Figure 3. The parameters are summarized in detail in Table 4. After the implantation of N^+^, the Nb peaks were slightly shifted to a lower angle and became broader. These results might indicate the implantation of N^+^ ions into the interstitial sites of the Nb lattices whereby the Nb lattices were expanded and the d-spacing increased. In addition, the grain refinement and partial amorphization might contribute to peak broadening [24]. The crystallite size was derived from the XRD patterns using the Williamson–Hall method. As can be seen in Table 4, the crystallite size tended to decreased with N^+^-implantation due to energetic ion bombardment. As previously reported, the Nb structure and physical properties were drastically changed depending on the implantation rate [25,26,27]. When the implantation rate was below 3 × 10^16^ N_2_/cm^2^, small defects including lattice strain or dislocation loop occurred. Subsequently, when the implantation rate exceeded 6 × 10^16^ N_2_/cm^2^, structural changes to the amorphous layer were initiated. Eventually, when the implantation rate reached 1 × 10^17^ N_2_/cm^2^, continuous amorphous layers were formed. In other words, the Nb layer changed its structure from crystalline to amorphous or a highly disordered phase when the implantation rate exceeded a certain level [27]. Based on the literature, since the implantation rate of the sample when 10 kV bias voltage (6.40 × 10^17^ N_2_/cm^2^) was applied exceeds the anticipated transition point to an amorphous phase, the amorphous layer should have formed on the surface. However, the formed amorphous layer was not very thick, and presented crystalline diffraction patterns with small, broadening peaks. In the case of 5 kV bias voltage, the amorphous layer was not expected to be formed since the implantation rate was below a certain level. Meanwhile, no additional peaks were observed in the XRD patterns such as Nb nitride. We therefore further performed an XPS analysis to investigate partial phase transformation. As shown in the N1s spectra (Figure 4), NbN, NbN_x_, and NbN_x_O_y_ peaks were detected, indicating that the implanted N^+^ ions randomly and partially formed nitride in the Nb matrix. The results also indicate that the intensity of the nitride peak was stronger at higher bias voltage. The detected NbN_x_O_y_ peak might arise from the formation of native oxide due to exposure in the air.

The phase characterization results suggested that a thin amorphous layer was formed on the surface and Nb nitride was partially formed in the Nb layer. The amorphous alloy is known to be a corrosion inhibitor, and often shows higher corrosion resistance than crystalline alloys at the same composition [28,29,30,31]. Furthermore, Nb nitride possesses good corrosion resistance even under aggressive environmental conditions, which have been widely studied for preventing metal corrosion [32,33,34,35]. Therefore, partial phase changes in the Nb layer might positively affect the corrosion behavior.

### 3.3. Microstructure

**Figure 5:**
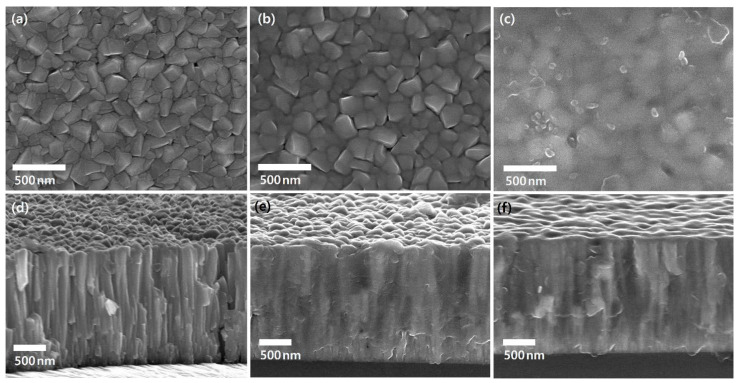
Surface and cross-section images: (**a**,**d**) as-deposited, respectively; (**b**,**e**) at 5 kV, respectively; and (**c**,**f**) 10 kV, respectively.

**Figure 6:**
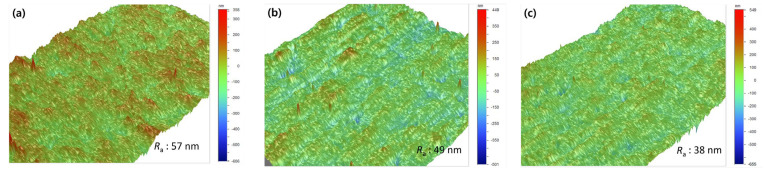
3D surface profiling images of (**a**) as-deposited Nb layer, (**b**) 5 kV, and (**c**) 10 kV.

The surface and cross-section of the as-deposited and N^+^-implanted Nb layers were observed by using electron micrographs, as shown in Figure 5. The FE-SEM images of the surface showed that N^+^-implanted Nb layers present truncated grain morphology due to ion bombardment which was absent in the as-deposited Nb layer. When 10 kV bias voltage was applied, a relatively nonuniform and blurred grain boundary was formed. The obtained results are consistent with the results of the surface roughness in Figure 6. Due to higher bias voltage, the crystalline structure is truncated and becomes smaller, resulting in a decrease in surface roughness. In the case of the cross-section, the as-deposited Nb layer presented a columnar structure with voids between columns; by contrast, in the N^+^-implanted Nb layers the interface between the columns became smoother and most of the voids were filled up. The obtained results demonstrated that the Nb layer was densified due to the bombardment of high energy ions.

To clarify the densification of the Nb layer, the defects on the surface and the inside of layers including voids and pinholes were examined. The critical passivation current density (CPCD) method was used to evaluate density and distribution of defects. The CPCD method electrochemically evaluates the defect area ratio (*R*_da_) using a current density at the active–passive transition region that is proportional to the exposed area of the substrate [36]. The calculated *R*_da_ pattern was as follows: as-deposited Nb (0.61 %) > 5 kV (0.57 %) > 10 kV (0.32 %). The *R*_da_ decreased with N^+^-implantation, and higher bias voltage led to further decrease. Therefore, a higher ion energy could contribute to densification of the structure, and it having less defects, which is consistent with microstructural observation. We assume that these microstructural changes would contribute to improving the corrosion behavior. The densified microstructure could prevent direct contact with the corrosive electrolyte, and decreased surface roughness could reduce surface area an exposed to the electrolyte.

### 3.4. Electrochemical Properties

**Figure 7:**
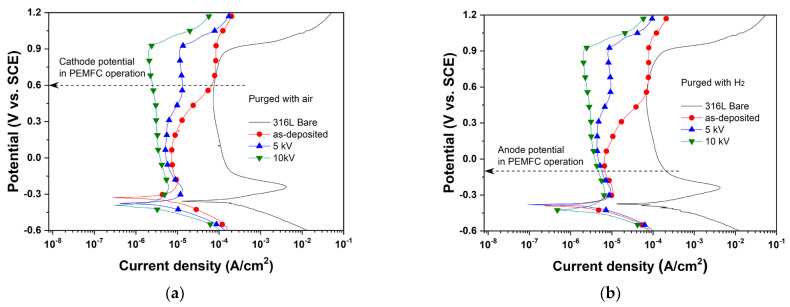
Potentiodynamic polarization curves of as-deposited Nb layer and N^+^-implanted samples with purged (**a**) air and (**b**) H_2_.

The potentiodynamic polarization test was performed under simulated PEMFC operating conditions (Figure 7, Table 5). The simulated cathode and anode conditions were described in the method section. As shown in Figure 7, the N^+^-implanted Nb layers exhibited a wide passive region compared to the as-deposited Nb layer and maintained passive state at both the cathode (0.6 V) and anode (–0.1 V) operating potentials. The corrosion current density (*i*_corr_) was obtained from the Tafel extrapolation and linear polarization, and was slightly decreased at the N^+^-implanted Nb layers. The *i*_corr, Tafel_ was determined from extrapolation between the *E*_corr_ with the anodic and cathodic Tafel slopes in the obtained polarization curve. The *i*_corr, Rp_ was calculated by the Stern–Geary equation using polarization resistance and tafel slopes. Both the obtained *i*_corr, Tafel_ and *i*_corr, Rp_ showed similar values and the same trend. Likewise, the passivation current density (*i*_pass_) was smaller than that of the as-deposited Nb layer. The lower value of *i*_pass_ implies a facile and fast transition to the passivation state. The polarization resistance (*R*_p_) was determined from linear polarization. In comparison with the as-deposited layer, the *R*_p_ of the N^+^-implanted Nb layers was increased. Moreover, when 10 kV bias voltage was applied, the *R*_p_ was more than twice that of the as-deposited Nb layer. Since the *R*_p_ implies tolerance to an oxidation reaction when an external potential is applied, it can be used to estimate corrosion resistance. Thus, the N^+^-implantation might contribute to greater protection against corrosion and enhanced tolerance to an aggressive oxidation environment. Although the corrosion potential was slightly decreased with N^+^-implantation, the corrosion and passivation current density and polarization resistance were improved with increasing bias voltage. In other words, even though the corrosion occurred a little earlier, the passive film was quickly formed and reached a passive state due to the N^+^-implantation effects.

Meanwhile, for the bipolar plate application, the corrosion behavior at the operating potential is highly significant. The N^+^-implanted Nb layers presented a smaller current density at the cathode (*i*_0.6 V_) and anode (*i*_−0.1 V_) operating potential when compared with that of the as-deposited Nb layer and bare 316L SS. In particular, the 10 kV applied sample showed the lowest value of current density in the cathode potential, that is one and two orders of magnitude smaller current density than that of the as-deposited Nb layer or bare 316L SS, respectively. Thus, these results suggest that the implanted N^+^ ions effectively protect the Nb layer and the substrate under an aggressive PEMFC operating environment.

**Figure 8:**
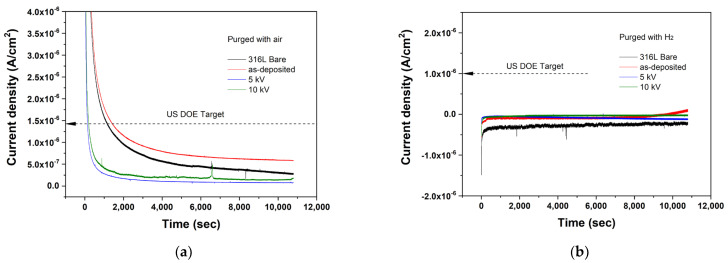
Potentiostatic polarization curves of the as-deposited Nb layer and N^+^-implanted samples with purged (**a**) air (constant 0.6 V) and (**b**) H_2_ (constant –0.1 V).

The bipolar plate is immersed in the corrosive electrolyte at both cathode and anode potentials during PEMFC operation. Thus, a durable corrosion resistance at the electrode potential is necessary. A potentiostatic polarization test performed under the simulated PEMFC operating conditions (Figure 8) showed that the higher initial current density of all the samples at the cathode (Figure 8a) rapidly decreased and stabilized to a lower value. However, a difference in the stabilization time was observed. The N^+^-implanted Nb layers stabilized within 2000 s, while the as-deposited Nb layer took more than two times longer for the current density to stabilize. This difference might correspond to the different redox reaction rates. A relatively fast redox reaction of the N^+^-implanted Nb layers resulted in the fast formation of a passive film on the surface [37]. Moreover, in the stabilized current density, the N^+^-implanted Nb layers exhibited a current density one order of magnitude smaller than that of the as-deposited Nb layer. In the case of the anode (Figure 7b), the polarization curves revealed a similar tendency to the cathode. Consequently, N^+^-implantation induced a more stable and lower current density in both cathode and anode, thereby satisfying the target set by the US department of energy (DOE).

The above results confirmed that the N^+^-implantation effectively modified the Nb layer, thereby improving electrochemical properties under the simulated PEMFC operating conditions. We regard the reason for such results likely to arise from the following effects. First, the bombardment of higher energy ions induced a densified Nb layer with fewer defects, which prevented direct contact between substrate and corrosive electrolyte. The synergetic effect between partially formed Nb nitride and the Nb matrix might contribute to additional improvement of electrochemical properties. In addition, tuning of the surface roughness by ion sputtering led to an increased electron work function and reduced the exposed area at the surface, thereby improving corrosion behavior [38,39,40]. It should also be considered that a thinly formed amorphous layer on the surface might contribute to surface passivation. Furthermore, in the present work, we investigated effective methods for modification of the protective layer. We found that this—in combination with reinforcement of substrate materials—will help further improve corrosion behavior of bipolar plate materials.

## 4. Conclusions

The deposited pure Nb layers were modified by the implantation of N^+^ ions, and the FE-SEM observation demonstrated that the higher ion energy could densify the Nb layer. The reduced defects inside the Nb layer effectively prevented the penetration of the corrosive electrolyte, resulting in improved corrosion resistance. Moreover, partially formed nitride in the Nb matrix further contributed to the improvement in corrosion resistance. In the polarization test, N^+^-implantation at 10 kV resulted in a current density one order of magnitude smaller than that of the as-deposited Nb layer. With respect to durability, N^+^-implanted Nb layers exhibited a current density one order of magnitude smaller with rapid surface passivation. Therefore, the N^+^-implantation effectively modified the deposited Nb layer to form an improved protective film for the metal bipolar plate. We believe that these results will provide new possibilities for surface modification and improvement of fuel cell performance, as well as other potential applications.

## Figures and Tables

**Table 1:** Chemical composition of 316L stainless steel.

Elements	Cr	Ni	Mn	Si	C	Mo	S	N	Ti	B	Cu	Fe
Wt%	16.62	10.1	1.35	0.43	0.018	2.06	0.028	0.046	0.01	0.01	0.34	balance

**Table 2:** Experimental conditions for Nb sputtering and N^+^ implantation.

**Nb sputtering**	Pressure (Pa)	0.666
	Gas flow (sccm)	10 (Ar)
	Target power (W)	600
	Bias voltage (V)	700
**N^+^ implantation**	Pressure (Pa)	0.14
	Gas flow (sccm)	5 (N_2_)
	Pulse width (μs)	3
	Bias voltage (kV)	5, 10

**Table 3:** Implanted ion range and concentration of TRIM simulation and SIMS analysis.

Bias (kV)	Ion Range (Å)		Implantation Dose (Ions/cm^2^)	Peak Nitrogen Concentration (Atoms/cm^3^)
	TRIM	SIMS		
5	95	57	2.78 × 10^16^	1.61 × 10^22^
10	158	140	6.40 × 10^17^	2.37 × 10^23^

**Table 4:** Conducted parameters of as-deposited Nb and N^+^-implanted samples from XRD patterns.

Bias (kV)	2θ (Degree)	*d*-Spacing (Å)	FWHM (Radian)	Crystallite Size (nm)
As-deposited Nb	38.40	2.342	0.020	17.00
5 kV	38.12	2.358	0.024	12.28
10 kV	38.16	2.356	0.025	11.76

**Table 5:** Corrosion parameters extracted from potentiodynamic polarization curves.

Sample	*E*_corr_ (V)	*i*_corr, Tafel_ (A/cm^2^)	*i_corr, Rp_* (A/cm^2^)	*i*_0.6V vs. SCE_ (A/cm^2^)	*i*_−0.1V vs. SCE_ (A/cm^2^)	*i*_pass_ (A/cm^2^)	*R_p_* (Ω cm^2^)
316L bare	−0.3374	1.33 × 10^−4^	1.67 × 10^−4^	4.95 × 10^−5^	1.31 × 10^−4^	3.64 × 10^−5^	61
As-deposited	−0.3278	1.26 × 10^−6^	2.08 × 10^−6^	4.78 × 10^−5^	7.88 × 10^−6^	7.69 × 10^−6^	5010
5 kV	−0.3688	1.19 × 10^−6^	1.43 × 10^−6^	1.31 × 10^−5^	5.67 × 10^−6^	5.08 × 10^−6^	6688
10 kV	−0.3746	1.08 × 10^−6^	1.23 × 10^−6^	2.52 × 10^−6^	4.66 × 10^−6^	1.99 × 10^−6^	11,457

## Data Availability

The data can be provided by authors on request.
